# Penis as a primary site of an extraskeletal Ewing sarcoma: A case report

**DOI:** 10.1097/MD.0000000000025074

**Published:** 2021-03-19

**Authors:** Dagmar Adamkova Krakorova, Jana Halamkova, Stepan Tucek, Ondrej Bilek, Jan Kristek, Tomas Kazda, Iva Staniczkova Zambo, Regina Demlova, Igor Kiss

**Affiliations:** a Department of Cancer Comprehensive Care Masaryk Memorial Cancer Institute, Zluty kopec 7; b Department of Cancer Comprehensive Care Masaryk Memorial Cancer Institute, Faculty of Medicine Masaryk University Zluty kopec 7, 656 53 Brno, Czech Republic And Department of Medical Ethics, Faculty of Medicine Masaryk University, Kamenice 25; c Department of Cancer Comprehensive Care Masaryk Memorial Cancer Institute, Faculty of Medicine Masaryk University Zluty kopec 7; d Department of Radiology, Masaryk Memorial Cancer Institute Zluty kopec 7; e Assoc. Prof. Department of Radiation Oncology Masaryk Memorial Cancer Institute Faculty of Medicine Masaryk University Zluty kopec 7; f Department of Pathology, St. Anne's University Hospital, Faculty of Medicine Masaryk University Pekarska 53; g Assoc. Prof. Clinical Trial Unit, Masaryk Memorial Cancer Institute Zluty kopec 7, 656 53 Brno, Czech Republic And Department of Pharmacology, Faculty of Medicine Masaryk University, Kamenice 25; h Assoc. Prof. Department of Cancer Comprehensive Care Masaryk Memorial Cancer Institute Faculty of Medicine Masaryk University Zluty kopec 7, Brno, Czech Republic.

**Keywords:** extraskeletal Ewing sarcoma, multidisciplinary treatment, penis

## Abstract

**Rationale:**

The Ewing sarcoma family of malignant tumors is a group of tumors characterized by morphologically similar round-cell neoplasms and by the presence of a common chromosomal translocation; Ewing sarcoma family of tumors typically occur in children and young adults between 4 to 15 years of age. The primary tumor usually originates in the bone, extraskeletal localization is rare.

**Patient concern:**

We present a case report concerning a 32-year-old male patient with a primary involvement of the penis.

**Diagnosis:**

The histopathology from the first penile biopsy showed a small-cell neuroendocrine carcinoma; however, that result was based on a sample obtained at a different facility than the Sarcoma Center, where the investigating pathologist did not have the adequate expertise. The patient then underwent a radical penectomy and a second reading of the histology was demanded after a radical penile amputation when Ewing sarcoma with R1 resection was confirmed.

**Interventions:**

The patient was referred to the national Sarcoma Center, where – using a multidisciplinary approach – the treatment was started with curative intent. However, it was preceded by a non-standard initiation of the therapy due to the poor primary diagnosis.

**Outcomes:**

The non-standard therapy at the onset of the disease caused a poor prognosis of an otherwise curable diagnosis. Despite all that, the patient survived for a relatively long time.

**Lessons:**

The treatment of sarcomas with atypical localizations should be conducted by an experienced multidisciplinary team in a center with experience in sarcoma treatment.

## 1 Introduction

**Figure 1:**
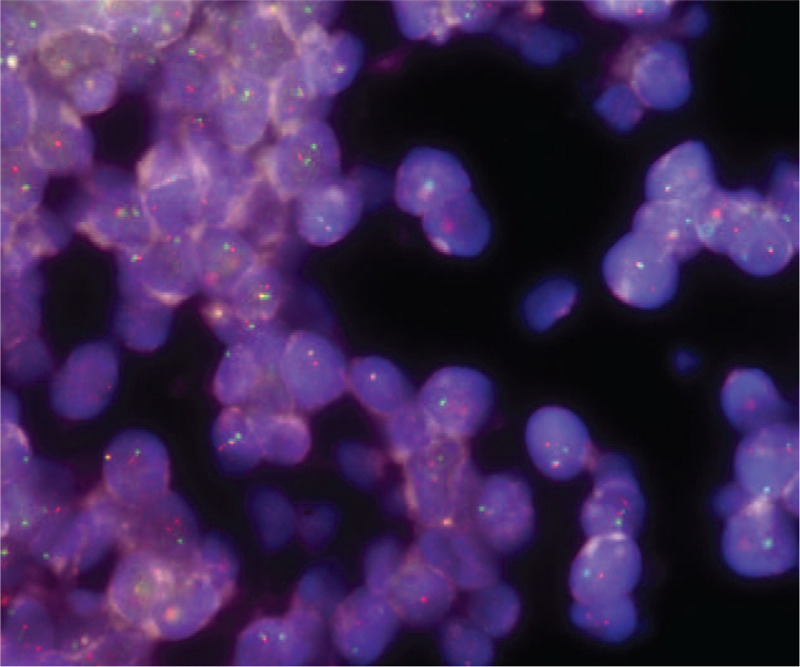
Positive EWS/FLI1 (t11;22)(q24;12) translocation (FISH). FISH = Fluorescence *in situ* hybridization.

Ewing sarcoma (ES) is a bone tumor typically occurring in children and young adults (4–15 years of age); it is a rare tumor with an incidence of 3 cases per 1 million per year. Ninety percent of patients are younger than 20 years. ES is of a neurogenic origin, in most cases originating in the bone marrow cavity of the pelvis or axial skeleton.^[1,2]^ Unlike in osteosarcoma, the involvement of long bones in the upper or lower limbs is less common. With its high biological activity, ES typically spreads early by the hematogenous route to the lungs, bones, bone marrow, but rarely into the central nervous system.^[3,4]^ Spread by lymphatic vessels is less common. Almost 25% of patients have become metastatic by the time of diagnosis.^[3,5]^ Direct spread and infiltration of surrounding tissues can be expected especially in cases with the involvement of flat bones and the chest wall. Primary extraskeletal origin is not common, representing 6% to 24% of cases.^[1]^ The tumors of the ES group are considered systemic diseases, with typical initial chemo- and radiosensitivity. Negative prognostic markers of this disease are: an extraskeletal localization, an axial skeletal involvement, a primary systemic spread, and a limited response to induction therapy. A common finding is a translocation between chromosomes 11 and 22 (t11/22, q24, q12). Other translocations (i.e. 7/22, 17/22) are also possible.^[6,7,8]^ The real-time polymerase chain reaction (RT-PCR) sensitivity is approximately 95%.^[9]^ The translocations lead to a juxtaposition of Ewing sarcoma genes on chromosome 22 and a Fms-like tyrosine kinase 1 gene on chromosome 11, and production of a chimeric protein with a transcription capability [Fig. 1].^[1,9,10,11]^ Additionally, new small-round-cell sarcoma entities with novel translocation have been recognized in recent years (*BCOR, CIC-FOXO4, CIC- DUX4* and other). In the 1960s and 70s, the standard treatment for local control was surgery and radiotherapy, with an overall 5-year survival rate of 22% of patients with localized disease.^[1]^ The addition of systemic chemotherapy has led to a remarkable improvement. With localized disease, the observed long-term survival of 65% to 70% children and 50% of young adults is up to 29 years.^[1,12,13]^ However, the prognosis of patients with a primary metastatic spread is much worse: up to 30% of patients reach 5-year survival in case of lung involvement, but less than 10% with a bone or bone marrow involvement.^[5,14]^ More than 20% of patients who were originally thought to have a localized disease, have bone marrow micrometastases detectable by RT-PCR.^[15]^ Negative and positive RT-PCR finding mean 2-year survival of 80% and 53% of patients, respectively.^[15]^ The conventional treatment can lead to a complete remission but it does not prevent the relapse of the disease in primary metastatic patients. The minimal residual disease, clinically undetectable, leads to relapse within 1 to 2 years after the treatment discontinuation. Since 2000, the treatment of localized or metastatic ES in Europe has been conducted according to the EURO- EWING 99 protocol.^[16]^ This protocol has been aimed at patients younger than 50 years. It consists of the vincristine, iphosphamide, doxorubicin, etoposide induction chemotherapy (using vincristine, iphosphamide, doxorubicin and etoposide), followed by stratification according to prognostic factors.^[16]^ Local therapy follows, surgery being preferred to radiation. The next step is consolidation therapy (chemotherapy +/− radiotherapy +/− high-dose chemotherapy). An updated version of the protocol includes Ewing 2008 and EURO Ewing 2012.^[17,18]^ Additionally, some protocol adjustments may be required considering the therapy tolerance and comorbidities.^[19,20]^

## 2 Case report

A 32-year-old male with no significant family history noticed a plum-sized lump on the left side of his penis in April 2010. Otherwise completely asymptomatic, he underwent an examination at a regional university hospital. Five months later, a tumorous infiltration arising probably from the urinary tube was diagnosed on CT.

**Figure 2:**
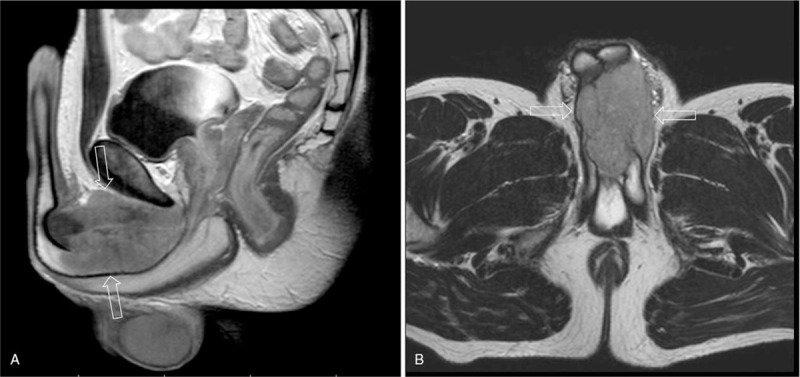
Initial findings of the penis base infiltration on MRI. A) T1-weighted, contrast-enhanced image in the sagittal plane showing a hypointense fusiform mass infiltrating corpora spongiosa with spicular extensions into the pubic fat pad and marked enhancement of the mass due to profuse vascularity. An aggressive, diffuse form of tumor growth is apparent. Note the relative sparing of the bulbospongiosus muscle and pubic bone. B) Same sequence, transversal plane documenting complete destruction of the anatomical structures and the macroscopic architecture of the tumor. MRI = magnetic resonance imaging.

**Figure 3:**
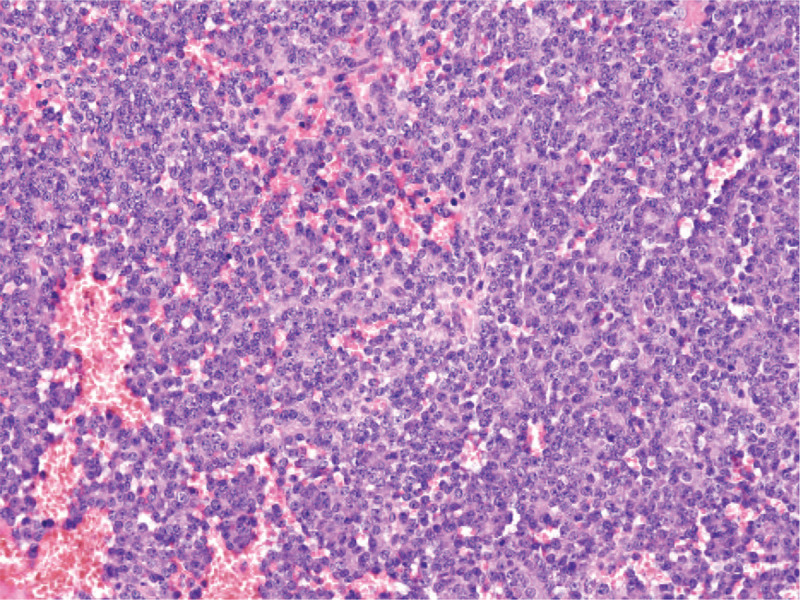
Histologically, Ewing sarcoma is composed of small cells with round hyperchromatic nuclei (x200 magnification).

Magnetic resonance imaging showed infiltration of both cavernous bodies (Fig. 2A,B). There was no pathologic lymphadenopathy in the pelvis or any infiltration of the bladder. The histopathology from the first penile biopsy showed a small-cell neuroendocrine carcinoma; however, that result was based on a sample obtained at a different hospital than the Sarcoma Center and the investigating pathologist did not have adequate expertise. A second histology reading was not demanded at that time. Bone scintigraphy, chest and brain CT did not confirm any metastatic disease. A total penile amputation, left side orchiectomy and perineostomy were carried out. The right testicle was transposed in the abdominal cavity. The definitive histology with a second reading confirmed an extraskeletal ES (Fig. 3) arising from the penis, with high proliferation and perineural propagation. The margins of resection were positive at the symphysis and the bulb of the penis. With these findings, the patient was referred for further treatment at the national Sarcoma Center. A quick restaging was conducted including trepanobiopsy. The assessment of the primary tumor showed a t11/22 translocation, the cytogenetic assessment of the bone marrow was negative. Using a multidisciplinary approach, treatment was started with curative intent, according to the EuroEwing 99 protocol and later Ewing 2008 protocol; however, with a nonstandard initiation due to the poor primary diagnosis. After the induction chemotherapy (6 cycles of vincristine, iphosphamide, doxorubicin, etoposide) had the patient no signs of disease. Consolidation chemotherapy was administered which contained 8 cycles of vincristine, dactinomycin, iphosphamide with concomitant radiotherapy of 45  Gray (Gy) in 25 fractions and 10.8 Gy in 6 fractions, respectively. The treatment tolerance was good, toxicity predictable, especially the grade 4 hematological toxicity in the induction phase. Maximal supportive care was provided. The treatment lasted for 10 months, ending one year after the diagnosis. The patient had been undergoing permanent antidepressant therapy, mainly due to the extent of initial surgical resection. The young man had been followed-up for 29 months with no signs of the disease. In October 2013, multiple lung metastases were diagnosed and induction chemotherapy combining irinotecan/temozolomide was started. In January 2014, a metastasectomy of all detectable pulmonary metastases was performed and the patient finished the chemotherapy treatment with an irinotecan/temozolomide combination in April 2014. In May 2014, the patient underwent high-volume lung irradiation with a dose of 12x1.5 Gy. After six months, a CT scan showed a relapse of the tumor in the dorsal mediastinum. In January 2015, the second thoracotomy was indicated. Marginal (R2) resection confirmed the recurrence of ES, with a 10% cell necrosis and high mitotic activity. Subsequently, metronomic chemotherapy modified with Combined Oral Metronomic Biodifferentiating Antiangiogenic Treatment including cyclophosphamide, celecoxib, vitamin D, and fenofibrate was started.^[21,22]^ In March 2015, the tumor in the mediastinum was irradiated with a dose of 40 Gy, showing a regression. The next chemotherapy with an etoposide/iphosphamide combination was started for the tumor progression in December 2015. After 6 cycles of chemotherapy, another progression of the tumor was determined and the patient was switched to fourth-line chemotherapy combining gemcitabine/docetaxel, which was terminated after 4 cycles due to further tumor progression. In still good overall condition, the patient started a new treatment with a topotecan/cyclophosphamide combination. The patient died due to rapid progression 3 months later, in February 2017.

## 3 Discussion

Extraskeletal localization of ES is rare; nevertheless, ES can be found in any location. So far, incidental cases of ES have been described of its occurrence in the vulva, vagina, uterus, ovaries, testicles, thyroid gland, bladder, kidneys, gallbladder, esophagus, stomach, and lungs.^[23–35]^ Based on the reviewed publications, only six cases of primary penile involvement of ES have been published (Pubmed 1970- Oct 2020). In 1999, Toh et al^[36]^ informed about a case of a 21-year-old patient with an ulcer lesion of the penile gland and a peritoneal mass, already metastatic to lungs at the time of the diagnosis, treated by the combination of iphosphamide, vincristine, doxorubicin, and dactinomycin, which resulted in regression after 3 months and a progression after 8 months of treatment. Another case was shown by Kilicaslan et al. in 2008^[37]^: A 19-year-old patient with a painless lump on the dorsum of the penis was treated with antibiotics repeatedly, later resected. No dissemination was found; nevertheless, a bilateral inguinal lymphadenectomy was performed with a negative result. The patient was treated with 4 cycles of iphosfamide, etoposide chemotherapy and chemotherapy vincristine/doxorubicin/cyclophosphamide chemotherapy, with a 20 Gy brachyradiotherapy combined with a 34 Gy external beam radiotherapy. The patient refused further treatment. Nine months later the patient relapsed in the lungs and the pleura. Subsequent salvage chemotherapy was not effective and resulted in a further progression 24 months after the diagnosis. In 1992 Jimenes-Verdejo et al. described a rare case of ES with metastatic involvement of the penis.^[38]^ Yonkenevich et al. published a case of a 65-year-old male with an extremely rare spindle-cell sarcoma.^[39]^ Because of the extensive unresectable nature of the disease, treatment with palliative chemotherapy iphosphamide and doxorubicin was chosen, with no response. Sharma et al presented a case of a 29-year-old patient with a penile ES spread to the lungs, treated with multi-agent chemotherapy including vincristine, doxorubicin, cyclophosphamide, etoposide, and iphosphamide followed by local radiotherapy and bilateral pulmonary metastasectomy. Adjuvant chemotherapy was applied using a combination of vincristine, doxorubicin, cyclophosphamide, topotecan, and cisplatin. Follow-up CT scans showed an increase in the size of lung metastases and local recurrence. Next, imatinib was used, followed by gefitinib, with no response.^[40]^ Ma et al also presented a case of a 28-year-old male with a primary penile ES.^[41]^ A pediatric Ewing sarcoma occurring in the genitourinary system has rarely been reported. He et al described four cases of pediatric patients, one involving a penile ES.^[42]^

ES is a rare chemosensitive and radiosensitive tumor which can occur in any location. The treatment procedure consists of combined induction chemotherapy, followed by local treatment with a preference for surgery over irradiation alone. Surgical treatment must include all initially affected tissues (i.e. not just a shrinking tumor after the chemotherapy) or must be supplemented by postoperative irradiation. After the local treatment, systemic chemotherapy is continued at 2 to 3 weekly intervals. The treatment of adult patients is based on the same principles and adaptations of the same treatment protocols as in children and adolescents. However, it is burdened with worse tolerance and higher toxicity, which is predictable and manageable using maximal supportive therapy, but justifiable due to high tumor chemosensitivity. The treatment of extraskeletal ES proceeds according to the same principles as in bone sites, with systemic chemotherapy administered in all cases and irradiation implemented in most cases.^[43]^

Our case report describes the long-term treatment of a curable rare disease where the prognosis of the patient was affected by non-standard management at the beginning of the diagnostic and treatment procedures. As a consequence, a young patient's quality-of-life was considerably impaired due to the amputation of the penis which could unfortunately be avoided.

The possibility of an atypical localization of rare tumors should be considered especially in young patients requiring centralized care and a multidisciplinary approach. The European Union is currently running the EURACAN project, where multidisciplinary teams are being set up at a European level, making it possible to consult rare tumors according to their location and histology type.^[44]^

## 4 Conclusion

In the cases of an atypical localization of malignant diseases and/or with the finding of rare histology, multidisciplinary management of the patients in an experienced center is highly recommended.

## Author contributions

**Conceptualization:** Dagmar Adamkova Krakorova, Jana Halamkova, Stepan Tucek, Jan Kristek, Tomas Kazda, Regina Demlova.

**Formal analysis:** Jana Halamkova.

**Funding acquisition:** Regina Demlova, Igor Kiss.

**Methodology:** Dagmar Adamkova Krakorova, Jana Halamkova, Regina Demlova.

**Project administration:** Dagmar Adamkova Krakorova, Jana Halamkova, Tomas Kazda.

**Resources:** Jana Halamkova.

**Supervision:** Tomas Kazda, Regina Demlova, Igor Kiss.

**Validation:** Regina Demlova.

**Visualization:** Jan Kristek, Iva Staniczkova Zambo.

**Writing – original draft:** Dagmar Adamkova Krakorova, Jana Halamkova, Stepan Tucek, Ondrej Bilek, Jan Kristek, Tomas Kazda, Iva Staniczkova Zambo.

**Writing – review & editing:** Dagmar Adamkova Krakorova, Jana Halamkova, Ondrej Bilek, Tomas Kazda, Regina Demlova, Igor Kiss.
